# Supplementary material for: Single-Cell RNA Sequencing on Formalin-Fixed and Paraffin-Embedded (FFPE) Tissue Identified Multi-Ciliary Cells in Breast Cancer
Source: Cells. 2025 Jan 29;14(3):197. doi: 10.3390/cells14030197 (PMC11816443; doi:10.3390/cells14030197)
Supplement: Supplementary file 1 [file cells-14-00197-s001.zip › Supplementary Figures.pdf]

Supplementary Figures

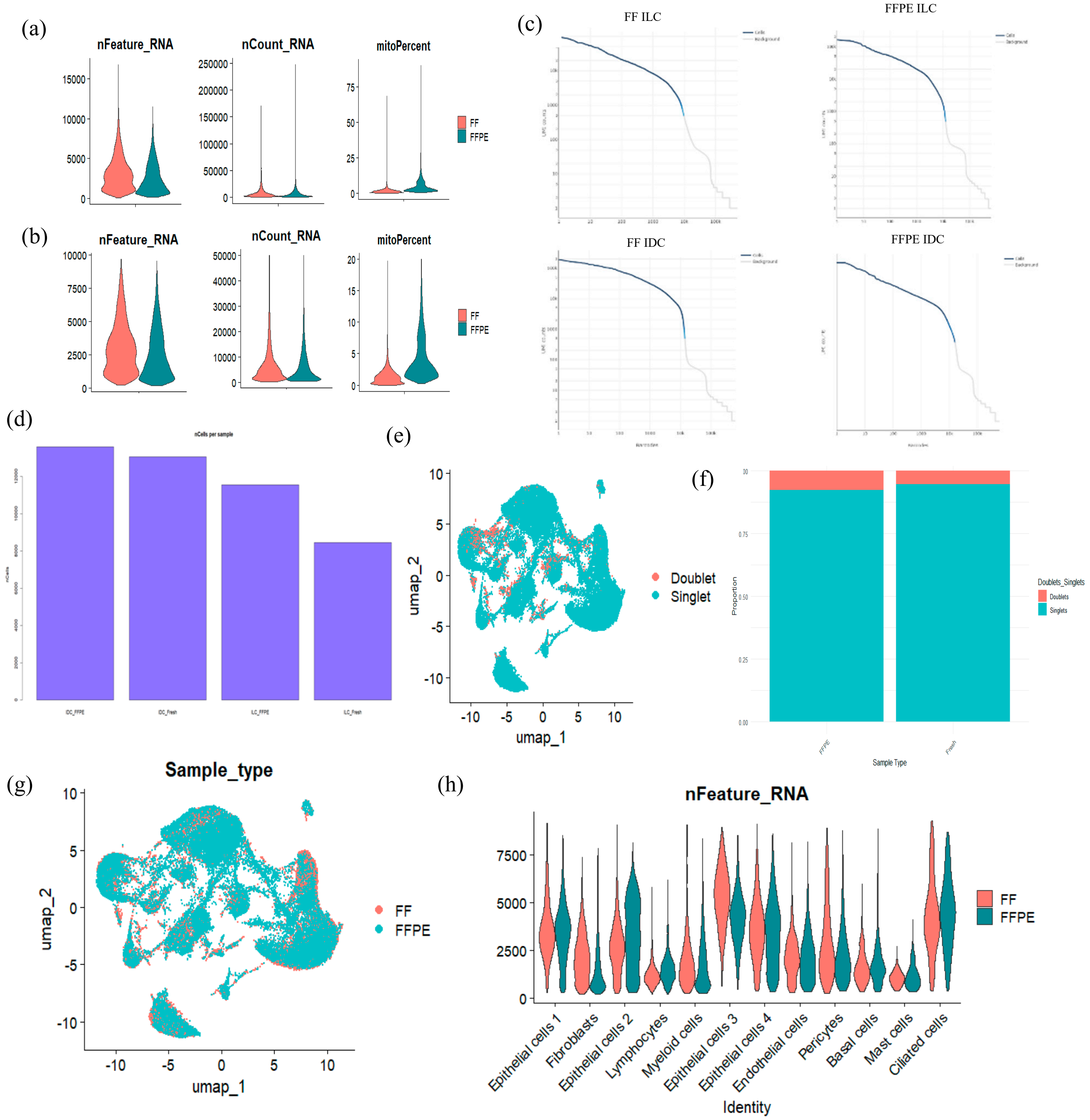

Figure S1. Quality control parameters. (a) Gene expression data, numbers of UMIs and fractions of mitochondrial reads per cell merged before filtering and colored by sample origin. (b) Gene expression data, numbers of UMIs and fractions of mitochondrial reads per cell merged after filtering for the quality parameters: >200 and <10,000 genes per cell, >250 and <5,000 UMIs per cell, fraction of mitochondrial reads lower than 20%. (c) Barcode Rank Plots showing the count of filtered UMIs mapped to each barcode. The color of the graph represents the local density of barcodes that are cell-associated. The plots demonstrated a high quality based on their profiles, all showing a distinctive "cliff and knee" shape. (d) Bar chart showing the number of cells per case after filtering. (e) UMAP plot of 43,599 individual cells from 4 samples generated from the top 15 principal components of all single-cell transcriptomes post-filtering, doublets removing and data integration, colored by singlets and doublets. (f) Bar plot showing singlets and doublets proportions in FFPE and fresh samples. (g) UMAP plot of 43,599 individual cells after doublet removal, colored by tissue origin. (h) Number of genes detected per cell in each cellular population in FFPE and fresh samples.

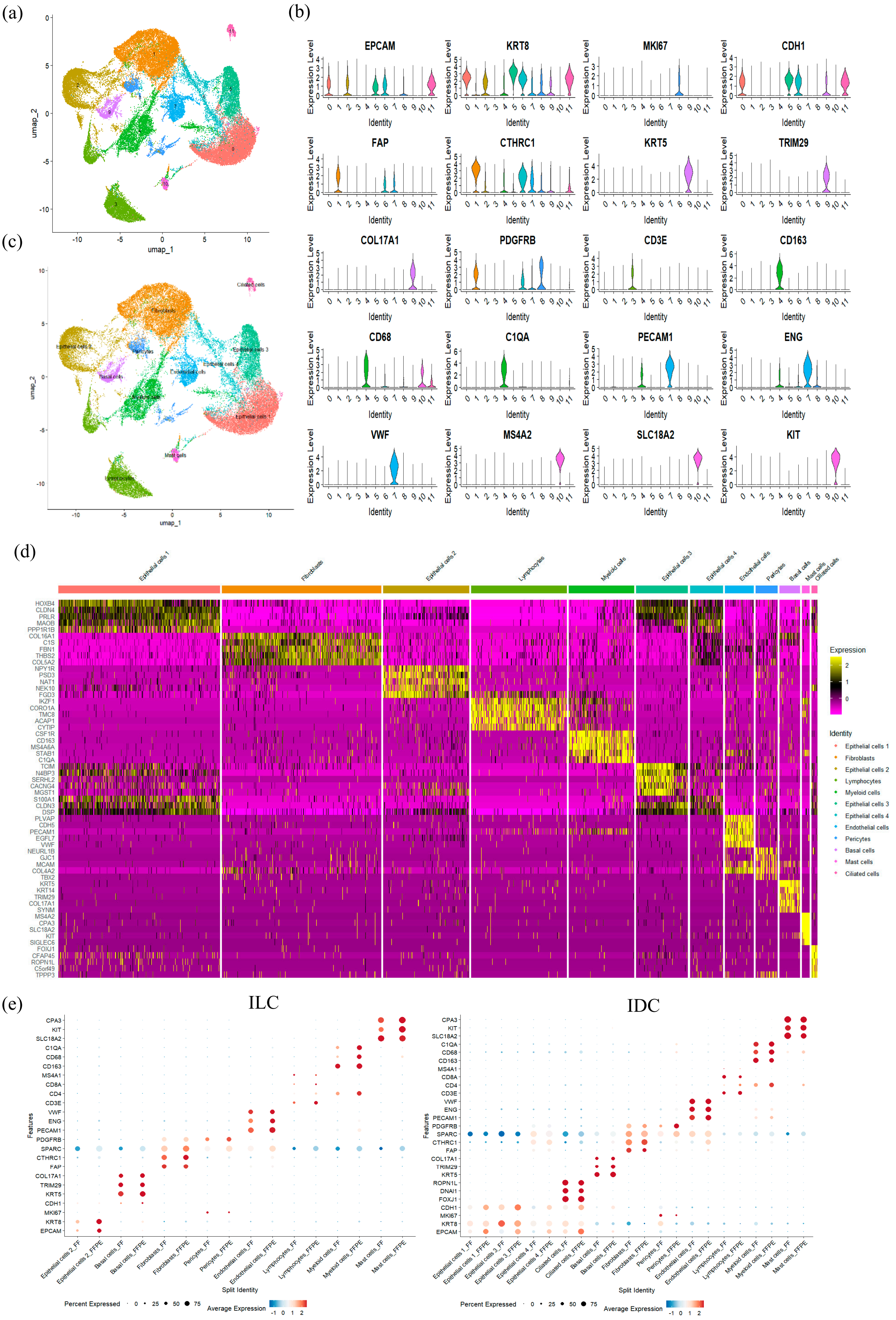
(a)
umap_1
umap_2
(b)
EPCAM
KRT8
MKI67
CDH1
FAP
CTHRC1
KRT5
TRIM29
COL17A1
PDGFRB
CD3E
CD163
CD68
C1QA
PECAM1
ENG
VWF
MS4A2
SLC18A2
KIT
Expression Level
Identity
(c)
Ciliated cells
Fibroblasts
Epithelial cells 2
Pericytes
Epithelial cells 3
Epithelial cells 4
Endothelial cells
Basal cells
Myeloid cells
Epithelial cells 1
Mast cells
Lymphocytes
(d)
Epithelial cells 1
Fibroblasts
Epithelial cells 2
Lymphocytes
Myeloid cells
Epithelial cells 3
Epithelial cells 4
Endothelial cells
Pericytes
Basal cells
Mast cells
Ciliated cells
HOXB4
CLDN4
PRLR
MAOB
PPP1R1B
COL16A1
C1S
FBN1
THBS2
COL5A2
NPY1R
PSD3
NAT1
NEK10
FGD3
IKZF1
CORO1A
TMC8
ACAP1
CYTIP
CSF1R
CD163
MS4A6A
STAB1
C1QA
TCIM
N4BP3
SERHL2
CACNG4
MGST1
S100A1
CLDN3
DSP
PLVAP
CDH5
PECAM1
EGFL7
VWF
NEURL1B
GJC1
MCAM
COL4A2
TBX2
KRT5
KRT14
TRIM29
COL17A1
SYNM
MS4A2
CPA3
SLC18A2
KIT
SIGLEC6
FOXJ1
CFAP45
ROPN1L
C5orf49
TPPP3
Expression
Identity
(e)
ILC
IDC
Features
Split Identity
Percent Expressed
Average Expression

Figure S2. scRNAseq annotation. (a) Clustered UMAP plot generated from the top 15 principal components of all single-cell transcriptomes post-filtering and data integration. (b) Markers for cell type annotation. (c) UMAP plot annotated according to the cell markers identified in Figure c. (d) Heatmap representing the top 10 most overexpressed genes (avg_log2FC > 1) in each cell population. (e) Dot plots showing the percentage of cells and the average gene expression levels of canonical markers for each cluster, analyzed separately in FF and FFPE samples for each histological type.

(a)

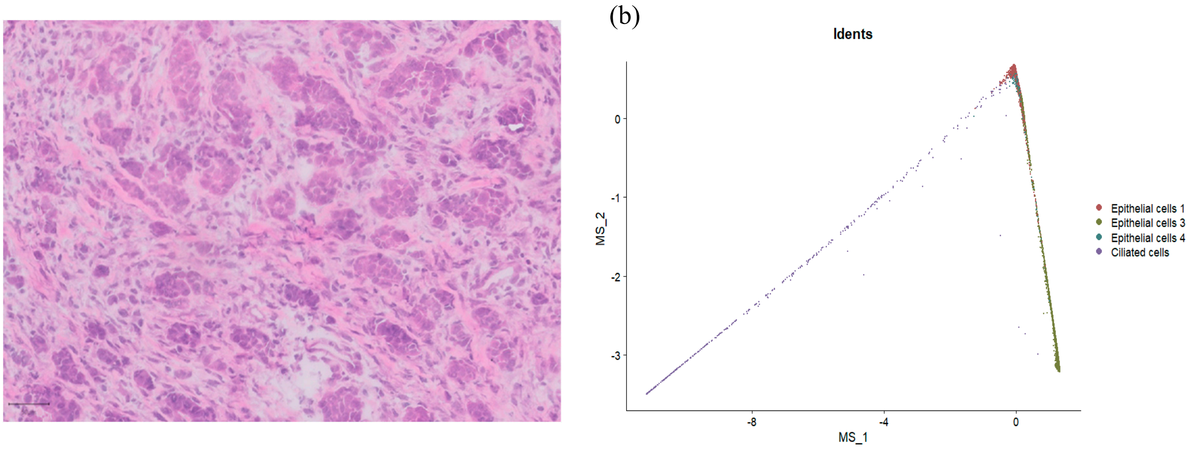

Figure S3. IDC cells. (a) IDC H&E staining (scale bar 50 μm). (b) Cell trajectory analysis of epithelial cells from IDC case.

(a)

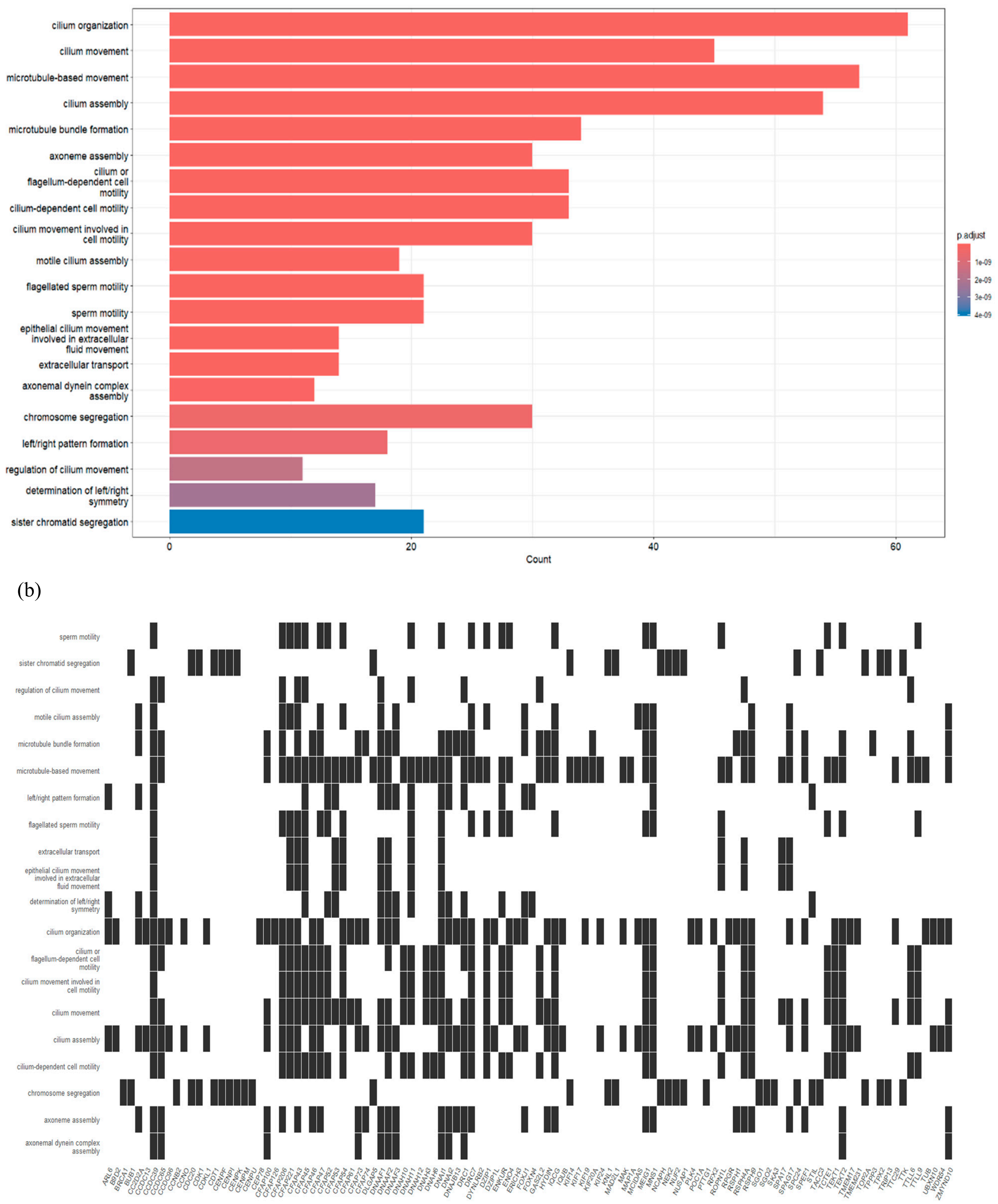

Figure S4. GO-enriched biological processes in multiciliated cells. (a) Bar plot displaying Go-enriched pathways significantly upregulated in multiciliated cells. (b) Heatmap showing the GO-enriched biological processes alongside the upregulated genes in multiciliated cells that contribute to these processes.

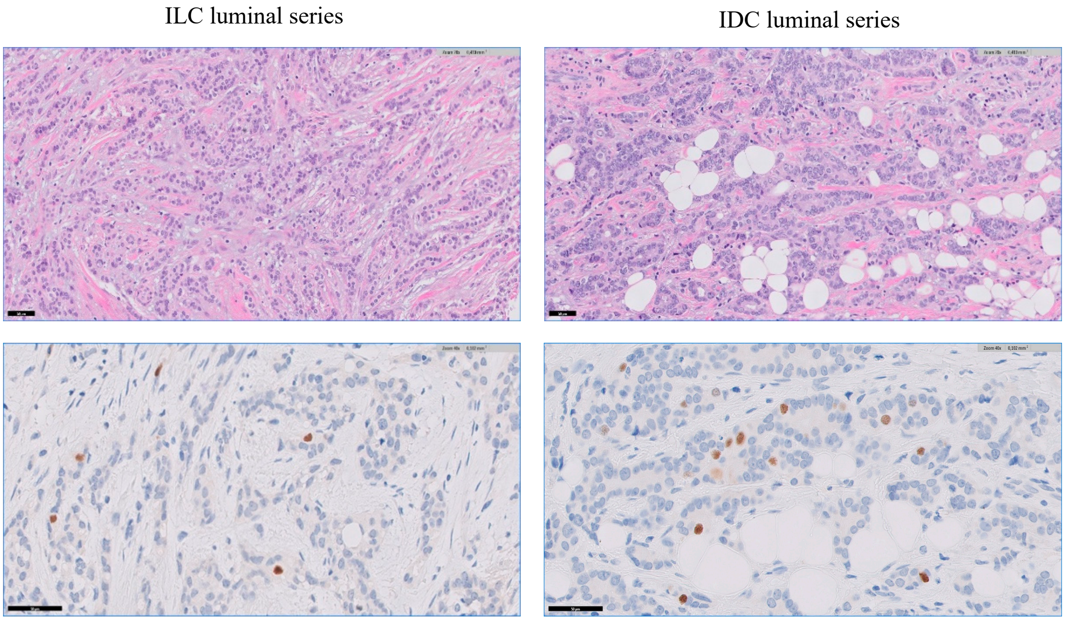

Figure S5. H&E staining, and FOXJ1 expression evaluation in one ILC and one IDC of the luminal breast carcinomas series (scale bar 50 μm).
